# Myco-generated and analysis of magnetite (Fe3O4) nanoparticles using Aspergillus elegans extract: A comparative evaluation with a traditional chemical approach

**DOI:** 10.1016/j.heliyon.2024.e31352

**Published:** 2024-05-18

**Authors:** Renjbar Muksy Mhammedsharif, Parwin Jalal Jalil, Nzar Piro, Ahmed Salih Mohammed, Peyman K. Aspoukeh

**Affiliations:** a Scientific Research Centre, Soran University, Soran, Kurdistan Region, Iraq; b Civil Engineering Department, Faculty of Engineering, Soran University, Soran, Kurdistan Region, Iraq; c Civil Engineering Department, College of Engineering, University of Sulaimani, Kurdistan Region, Iraq

**Keywords:** Myco-synthesis method, Fe_3_O_4_ NPs, Reducing agent, Capping agent, Stabilizing agent, *Aspergillus elegans* extract

## Abstract

In the past few years, nanotechnology has emerged as one of the most interesting and cutting-edge research areas across all disciplines. Nanotechnology allows progress in all science fields to make novel materials and industry-different devices. Generally, nanoparticle synthesis methods are chemical, physical, and biological. The chemical and physical techniques use potentially harmful compounds, and the expense of these processes renders them unsuitable for nanoparticle synthesis. In light of this, it needs development strategies that are sustainable, economical, and eco-friendly viable. Through, biosynthesis, nanoparticles can overcome these disadvantages. One of the biological strategies is the myco-synthesis method, which connects the fields of mycology and nanotechnology. In this study, magnetite (Fe_3_O_4_) NPs have been synthesized using a myco-synthesis method by selecting *Aspergillus elegans* as a fungal species. Two extracts were used, growth medium and an aqueous extract. A comparative analysis between nanoparticles synthesized through myco-synthesis and those produced using conventional chemical methods has been conducted to substantiate the significance of the biological approach. The results of this study unequivocally establish that myco-synthesized nanoparticles exhibit superior and enhanced characteristics compared to those synthesized through chemical means, as ascertained through a comprehensive array of characterization techniques employed throughout the investigation. This contrast is observable in terms of the aggregation state, the existence of capping and stabilizing agents enveloping the nanoparticles, their magnetic and thermal attributes, and the enduring stability of these nanoparticles. These results highlight the significant promise of employing phytochemicals extracted from Aspergillus elegans as a highly suitable option for the biofabrication of Fe_3_O_4_ nanoparticles.

## 1 Introduction

Nanotechnology has attracted exceptional attention due to its distinct physicochemical features and widespread application in several sectors in recent years. Several scientists and economists observed a dramatic growth in the use of nanomaterials to meet the rising demand in many application sectors, especially with the creation of novel nanomaterials Maurer-Jones et al., 2013 [1]. Nanoparticles (NP) are typically clumps of atoms that vary in size from one to one hundred nanometers. It is well known that a metal NP's dimensions, form, composition, crystallinity, and structure are the primary factors influencing its characteristics Sun and Xia, 2002 [2]. The unique crystallographic nature of NPs causes them to have a greater surface area, leading to a greater degree of reactivity. This is also true despite NPs having a very small physical structure [3]. NPs may be synthesized from various materials and formed into various forms. In the past few years, the subject of nanotechnology has emerged as a field of study that may use in optics in a wide variety of contexts [4], electronics [5], catalysis [6], biomedical sciences [7], energy science [8], agriculture [9], and environment among other industrial sectors [10].

Synthesis of nanomaterials may often be broken down into two primary categories: top-down and bottom-up. To get thin-layer crystals using a top-down strategy, it must frequently disrupt the van Der Waals forces holding the stacked bulk components together. The bottom-up synthesis technique for nanoparticles might entail the formation of ionic or covalent bonds. The top-down strategy often requires massive amounts of energy inputs and leaves a significant footprint on the environment. Manufacturing nanoparticles used to clean up the environment shouldn't result in any significant side effects [11]. Top-down approaches include laser ablation, ion sputtering, and mechanical milling [12]. Although top-down procedures are generally simpler to carry out, this method cannot be used to execute the process of performing on extremely tiny-sized particles. This method of producing nanoparticles comes with several significant challenges, the most significant of which is a change in the physicochemical characteristics of the nanoparticles and their surface chemistry. Bottom-up approaches by aggregating atoms, molecules, or smaller particles may accomplish the formation of nanoparticles. The production of the ultimate nanoparticle begins with the development of nanostructured building blocks of nanomaterials, which then proceed by integration to make the final stage nanoparticle. Bottom-up approaches include physical, chemical vapor deposition sol-gel, hydrothermal, spray, and laser pyrolysis [13]. In both physical and chemical techniques of fabricating nanoparticles, using chemicals and high temperatures and pressures negatively affects the surrounding environment. The biological method suited to the synthesis of nanoparticles involves the use of plant products and microorganisms. Biological techniques are among the most ideal for the creation of nanoparticles for the cleanup of contaminants because they are the most economically efficient, environmentally friendly, and adaptable approaches, and they also eliminate the use of harmful chemicals, high pressure, energy, and temperature [14].

As a direct consequence of this fact, scientists working in nanoparticle fabrication and construction have begun looking to biological systems for ideas. This should not come as a surprise, considering that it has been demonstrated that a variety of single-cell and multi-cell organisms, are capable of producing inorganic materials either within or outside of their cells. The synthesis of magnetite nanoparticles by magnetotactic bacteria is one well-known example of the production of inorganic materials by microorganisms [[15], [16], [17]]. S-layer bacteria are responsible for the production of gypsum and calcium carbonate layers. Diatoms are responsible for the synthesis of siliceous materials. The insights gained from studying nature have been essential in the creation of biomimetic strategies for the production of cutting-edge nanomaterials [[18], [19], [20], [21], [22]].

Fungal nanotechnology, also known as Myconanotechnology, is a made-up phrase that was first used in 2009 by an Indian researcher by the name of Rai M. (Myco is short for fungi, and nanotechnology refers to the manufacture and usage of materials in the 1–100 nm size range). Fungi produce nanoparticles used in various applications, most notably in the biomedical, environmental, and agricultural industries [[23], [24], [25]].

Mycogenic synthesis of nanoparticles is an important component of myco-nanotechnology. Due to the broad spectrum and variety of fungi, this process gives rise to an exciting new and practically applicable interdisciplinary science with considerable outcomes [26]. Myco-synthesized nanoparticles have seen significant usage in various applications, including the detection and elimination of pathogenic agents, the purification of wastewater, the preservation of food, the production of nematicides, and many more. Mycogenic nanoparticles, generated by a wide variety of fungal species, can be used in several agricultural applications to boost crop yield by enhancing growth and providing resistance to diseases. In addition, this will increase the toxicity of chemical pesticides, insecticides, and herbicides against plant ecosystems [27]. Pathogens responsible for infectious illnesses in humans have been demonstrated to be inhibited effectively by nanoparticles mediated by fungi, particularly when it comes to infections considered multi-resistant to conventional antibacterial drugs [28]. In the realm of heavy metal biosorption from wastewater contaminants, fungi-based nano sorbents are also a creative research direction that has been taken [29].

Iron oxides consist of anionic arrangements that commonly exhibit cubic or hexagonal symmetry. The spaces between particles are partially occupied by trivalent or divalent iron, with a prevalence of octahedral coordination in the form of FeO_6_. However, there have also been reports of tetrahedral coordination in the form of Fe_3_O_4_ [30]. Iron oxide nanoparticles have differentiated themselves from bulk materials as a direct consequence of the features of iron oxide that are inversely related to the size and morphology of their structure [31]. Several different types of iron oxides may be found in nature, including hematite (-Fe_2_O_3_), magnetite (Fe_3_O_4_), and maghemite (-Fe_2_O_3_) [32]. Because of its remarkably rigid framework and higher concentration of divalent iron ions, Fe_3_O_4_ has the highest level of magnetism compared to the other iron oxides [33]. While, Maghemite is a mixture of the structure and content of hematite and magnetite; it has a color that may be described as reddish-brown, and it is formed in the ground as a consequence of magnetite being exposed to the elements. Hematite is the oldest type of iron oxide discovered in scientific circles and is also referred to as ferric oxide [34].

Iron oxides are often considered to be the most explored magnetic nanoparticles due to their lack of toxicity and overall compatibility with living systems [35]. In addition to these characteristics, the capacity of iron oxide to be separated and its magnetic properties are also beneficial to the recycling process. This why the magnetic nanoparticles are chosen to be synthesized and used in different fields [36]. Some of the physical and magnetic properties of iron oxides are shown in Table 1.

**Table 1:** Physical properties of iron oxides [37].

Properties	Magnetite	Maghemite	Hematite
Molecular formula	Fe_3_O_4_	γ-Fe_2_O_3_	α-Fe_2_O_3_
Crystallographic structure	Cubic	Cubic or tetrahedral	Rhombohedral or Hexagonal
Lattice Parameter (nm)	a = 0.8396	a = 0.8347	a = 0.5034
Color	Black	Reddish-Brown	Red
Magnetic Status	Ferrimagnetic or superparamagnetic	Ferrimagnetic or superparamagnetic	Weakly Ferromagnetic
Hardness	5.5	5	6.5
Density (g/cm^3^)	5.18	4.87	5.26

The synthesis of nanoparticulate magnetite (Fe_3_O_4_) and greigite (Fe_3_S_4_) by bacteria has been investigated deeply; nevertheless, the biochemical mechanism has not yet been completely understood. This study demonstrated that nanoparticulate magnetite may be generated extracellularly at room temperature by presenting the fungus *Aspergillus elegans* extract with combinations of the salts iron chloride (FeCl_2_) and iron chloride (FeCl_3_) [38,39]. Using fungal biomass and secreted enzymes as bioreducing and capping agents aligns with green chemistry principles. Fungi can mediate the nucleation and growth of nanoparticles, resulting in various shapes and sizes ranging from spheres and rods to triangles and hexagons [[40], [41], [42], [43], [44], [45]].

Microorganisms that survive in environments with high atmospheric pressure, acidity, and temperature are promising candidates for creating IONPs. Fungi have been reported as a novel and effective candidate for the environmentally friendly production of IONPs. This is because fungi are simple to handle, have a faster growth rate, and require low-cost maintenance, making them ideal organisms for the environmentally friendly synthesis of nanoparticles. They can survive agitation in bioreactors because of their filamentous form, and they can produce massive quantities of extracellular enzymes in a very short time when incubated [46].

Fungal cells possess transport mechanisms that allow them to remove metal ions from the surrounding environment. These ions are often present as metal salts dissolved in the growth medium. The uptake is driven by ion concentration gradients and facilitated by ion channels and transporters. Once inside the cells, metal ions are subjected to reduction processes. Fungi employ various intracellular reducing agents, such as NADH (nicotinamide adenine dinucleotide) and NADPH (nicotinamide adenine dinucleotide phosphate), which are cofactors involved in cellular metabolic reactions. These reducing agents donate electrons to the metal ions, converting them into neutral atoms or small clusters [47].

Myco-nanoparticles often exhibit excellent biocompatibility due to biomolecules on their surfaces. This characteristic enables functionalization through bioconjugation, enabling the attachment of ligands for targeted drug delivery or specific bio-recognition. The physical and chemical properties of myco-nanoparticles can be controlled by modulating the fungal growth conditions and synthesis parameters. This tunability facilitates the design of nanoparticles with desired properties, such as enhanced plasmonic resonance, improved magnetic behavior, or tailored catalytic activity [[48], [49], [50]].

The colonies of *A. elegans* were bright yellow and prolific. Fully mature, septate, branching, hyaline mycelium. Each cell has several nuclei. The conidiophores were very elongated, frequently including a foot cell that was either straight or flexuous and swelled into a globular vesicle at the tip. Branches more or less clenched cover the vehicle's surface. The organism had globose, catenulate, dry, echinulate, or biseriate conidial heads, and its conidia were yellow to ochre in hue, while its sclerotia were a dark brown tint. *A. elegans* had the greatest mycelial development (95.33 mm) on the PDA medium. According to the results, *A. elegans* grows most rapidly at a temperature of 30 °C. Mycelial growth of *A. elegans* was greatest (88.25 mm) at pH 7, medium at pH 8 and pH 6, and least at pH 9 [51,52]. According to Kumla et al. [53], the following metabolites were recovered from *A.elegans*: clavatol, sitostanol, vioxanthin, xanthomegnin, viomellein, rubrosulphin, rubrosulphin diacetate, viopurpurin, ochratoxin A, ochratoxin A methyl ester, ochratoxin B, and ochratoxin.

The extract derived from *A. elegans* is endowed with phytochemical constituents, notably flavonoids. These compounds play a pivotal role in mitigating nanoparticle aggregation and facilitating the stabilization of the syntheszied nanoparticles. There is no study to create nanoparticles with this species of fungi. Hence, the novelty of this study was to create nanoparticles with *A.elegans* and compare the performance of nanoparticles using *A.elegans* extract with the NPs prepared from the conventional chemical method. This investigation aimed to synthesize nano magnetite, specifically Fe3O4 nanoparticles, employing extracts from A.elegans. These nanoparticles were subsequently subjected to a comparative analysis with magnetite nanoparticles prepared through the conventional chemical method, following the protocol outlined by Bharde et al. [54], albeit with certain modifications. This study aimed to enhance the quality of magnetic nanoparticles through a biologically mediated approach and evaluate their characteristics concerning conventionally synthesized counterparts.

## 2 Method and material

### 2.1 Materials

**Fig. 1:**
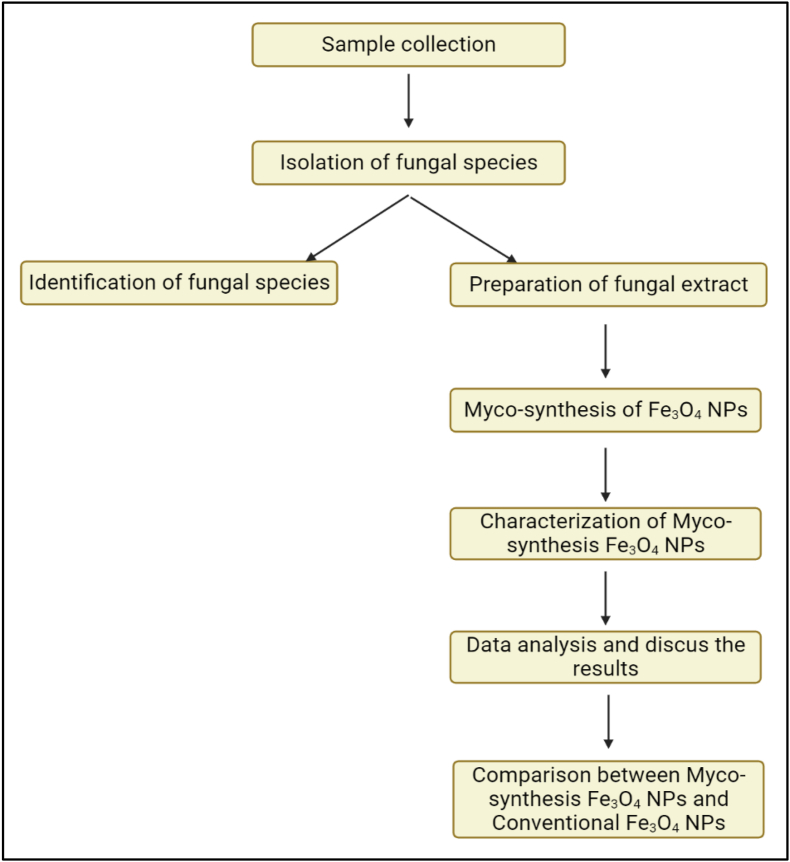
Flow chart of the experimental procedure of the study.

Iron chloride tetrahydrate (FeCl_2_·4H_2_O) and iron chloride hexahydrate (FeCl_3_·6H_2_O) were procured from Biochem Chemopharma Company, France, and utilized as precursors in the fabrication of magnetite nanoparticles. Additionally, sodium hydroxide (NaOH) was also acquired from Biochem Chemopharma Company. Specimens for the isolation of *A.elegans* were sourced from a landfill site in Soran city, located within the Kurdistan region of Iraq, at geographical coordinates (36.6246699 latitude and 44.5254009 longitude). Fig. 1 shows the flowchart of the study.

### 2.2 Isolation of *A.elegans*

**Fig. 2:**
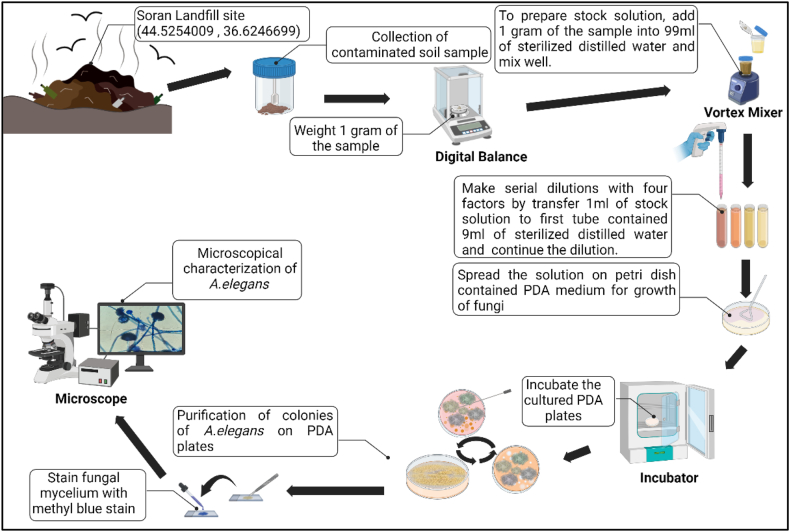
Isolation and identification of fungal species from landfill. The image was created with BioRender.com.

The isolation of fungal species from soil contaminated with municipal waste material was achieved using the dilution method, followed by transferring 100 μl from diluted samples onto Potato Dextrose Agar (PDA) medium. Afterthat, The culture plates were put into inucubator for 5–7 days at 30 °C. Next, individual colonies were subcultured onto pure PDA plates to obtain pure cultures [52]. The isolated fungi were identified through a process that involved morphological characterization of their colony growth patterns and microscopic examination of their fungal structures [[55], [56], [57], [58], [59],^60^]. The microscopic assessment of the isolated fungi was conducted using methylene blue staining techniques and light microscopy (model; Leica DM2700 P, Germany) (Fig. 2).

### 2.3 Preparation of fungal culture filtrate

**Fig. 3:**
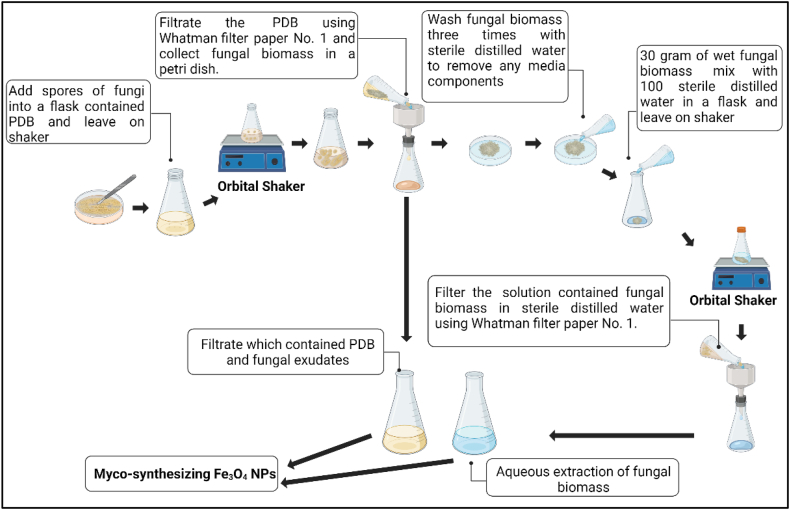
Preparation of fungal extract for biosynthesis of Fe_3_O_4_-NPs. The image was created with BioRender.com.

The fungal strain was cultivated in a Potato Dextrose Broth (PDB) medium consisting of 200 ml of potato extract and 20g of glucose dissolved in 1 L of distilled water, maintaining a pH of 7. The culture was incubated at 28 °C for 15 days. After this incubation period, the culture medium underwent filtration using Whatman filter paper no. 1. A volume of 100 ml of the filtered medium, referred to as the "growth medium," was aseptically transferred into a sterilized Erlenmeyer flask to synthesize Fe_3_O_4_ nanoparticles. Fifteen grams of the harvested fungal biomass were then separately re-suspended in 100 ml of sterile distilled water and subjected to further incubation at a temperature of 28 ± 2 °C while agitating at 150 rpm for 48 h. Subsequently, cell-free filtrates, also known as aqueous extracts, were obtained by separating the fungal biomass through filtration using Whatman filter paper No. 1. A volume of 100 ml of these aqueous extracts was employed for the biosynthesis of Fe_3_O_4_ nanoparticles as shown in Fig. 3.

### 2.4 Magnetite NPs synthesis using *A. elegans* extract

This study employed a reliable and consistent method to synthesize magnetite nanoparticles. To initiate the process, 100 ml of growth medium and 100 ml of aqueous extract were added separately drop by drop to a 50 ml water solution containing 2 g of FeCl_3_·6H_2_O and 1 gam of FeCl_2_·4H_2_O. The mixtures were continuously incubated at 50 °C with stirring for 30 min. The transformation of the color of the mixture to black signed the successful production of magnetite nanoparticles.

Additionally, 1 N NaOH solution was incorporated into the mixture above. Furthermore, the solution was consistently heated to 80 °C until the water had evaporated, resulting in the participation of nanoparticles at the bottom of the container.

**Fig. 4:**
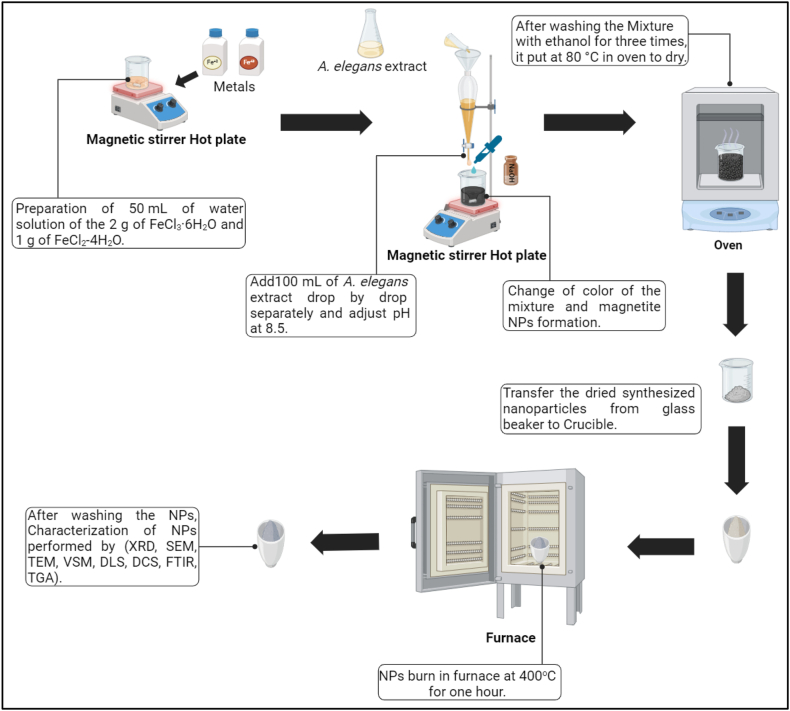
Myco-synthesized Fe_3_O_4_ NPs using growth medium and aqueous extract of *A.elegans.*

Subsequently, the participating particles were subjected to three rounds of washing, two rounds with double-distilled water and then with ethanol, in order to purify the synthesized nanoparticle from all contaminants. The synthesized nanoparticles were then subjected to a heat treatment in a furnace at 400 °C for 1 h, as illustrated in Fig. 4.

### 2.5 Characterization of myco-synthesized NPs

A variety of distinct techniques and instruments were employed in the comprehensive characterization of the Fe3O4 nanoparticles produced through myco-synthesis. X-ray diffraction (XRD) analysis was executed using the Panalytical X'Pert3 Powder instrument, which featured a diffractometer system (XPERT-PRO) and employed Cu ka radiation. The diffraction pattern was recorded over a 2θ range spanning from 5° to 70°, utilizing a 2θ step scan of 0.010, with each step counted for 0.5s. The generator operated at 45 kV and 40 mA [61,62]. The resulting micrograph was cross-referenced with the Joint Committee on Power Diffraction Standards Library to ascertain the crystalline structure of the nanoparticles. Moreover, the nanoparticle sample underwent lyophilization and underwent further scrutiny. Morphological attributes were examined through scanning electron microscopy, specifically utilizing the FEI Model QUANTA 450. Zeta potential measurements were performed using a Nano ZS90 Zeta sizer from Malvern Instruments, incorporating a He–Ne laser (633 nm, 5 Mw). Dynamic light scattering (DLS) analysis was conducted utilizing the same Nano ZS90 Zeta sizer. Additionally, the analytical repertoire encompassed Differential Scanning Calorimetry (DSC), Vibrating-Sample Magnetometry (VSM), Thermogravimetric Analysis (TGA) with the TGAQ500 V20.13 Build 39 instrument sourced from TA Instruments Co., USA, Transmission Electron Microscopy (TEM), and Fourier Transform Infrared (FTIR) spectroscopy.

## 3 Results and discussion

### 3.1 Isolation and identification of *Aspergillus elegans*

**Fig. 5:**
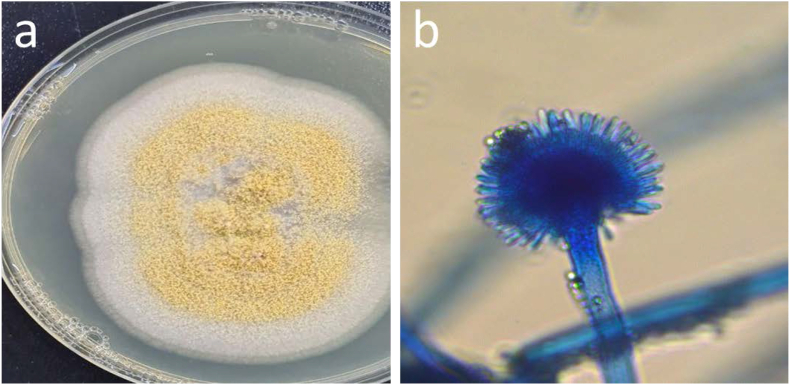
Macroscopic and microscopic characterization of fungal species a) Colony of *A.elegans* on PDA medium b) Sporangium and sporangiophore of *A.elegans* under a light microscope.

The isolated fungi, *Aspergillus elegans* were characterized based on the morphology of the colonies (Fig. 5a) and microscopic characterization of the mycelium, sporangium, and conidia (Fig. 5b). The isolated fungus conformed to belong Aspergillus genus; it was correctly *Aspergillus elegans*.

### 3.2 XRD analysis of myco-synthesized NPs

**Fig. 6:**
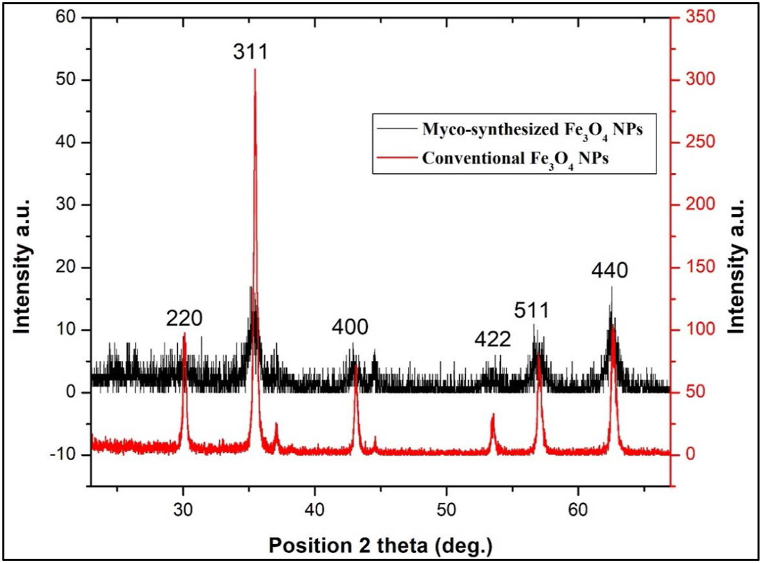
XRD analysis for Myco-synthesized Fe_3_O_4_ NPs from the growth medium and the magnetite NPs prepared from the conventional chemical method.

**Fig. 7:**
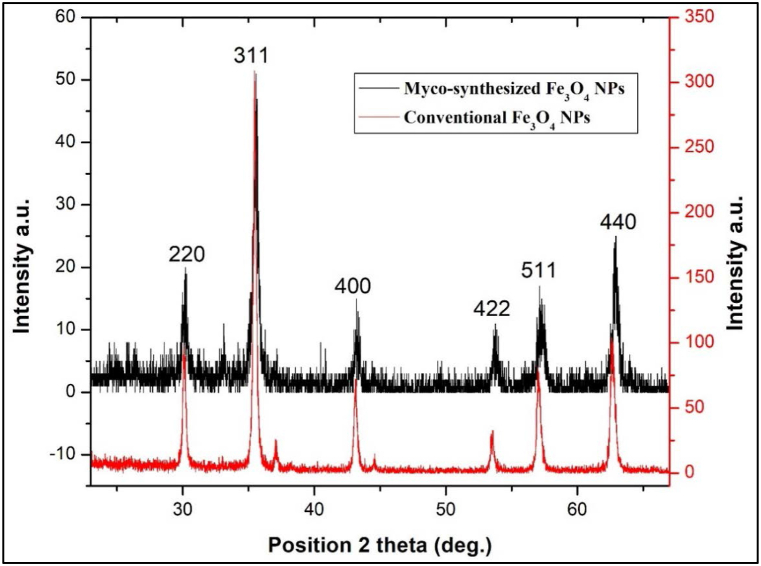
XRD analysis for Myco-synthesized Fe_3_O_4_ NPs from aqueous extract and the magnetite NPs prepared from conventional chemical method.

X-ray diffraction (XRD) stands out as one of the most commonly employed techniques for nanoparticle (NP) characterization. The device used in this study featured a Cu anode, a wavelength of 0.154 nm, a maximum power of 2.2 kW, and a long, fine-focus ceramic tube. Fig. 6, Fig. 7 present the XRD powder diffraction patterns of Fe3O4 NPs, synthesized via both biosyntheses using A.elegans and the conventional chemical method. Notably, the diffraction peaks observed for the myco-synthesized Fe_3_O_4_ NPs exhibit broadening, which can be attributed to the small crystallite size. This phenomenon indicates a higher level of phase purity in the product. The cubic structure of Fe_3_O_4_ served as an index to all the identified diffraction peaks, which are all connected to Fe_3_O_4_ (JCPDS no. 19–629) Fig. 6, Fig. 7. The XRD pattern revealed the peaks at 30.17°, 35.62°, 43.21°, 53.75°, 57.27°, and 62.92° for my-synthesized Fe_3_O_4_ NPs from aqueous extract of fungi and 35.28°, 43.08°, 44.51°, 56.72°, and 62.46° for myco-synthesized Fe_3_O_4_ NPs from the growth medium solution of fungi and 30.08°, 35.46°, 43.1°, 53.53°, 57.02°, and 62.7° for the magnetite NPs prepared from the conventional chemical method. The identification of the particles as Fe_3_O_4_ with a cubic inverse-spinel structure is substantiated by the observed 2θ values corresponding to specific Bragg's reflections for FCC structures, namely (220), (311), (400), (422), (511), and (440) planes. Moreover, the average particle sizes were determined to be 21.18 nm and 13.96 nm for Fe_3_O_4_ nanoparticles synthesized through myco-synthesis using the growth medium and aqueous extract, respectively. In contrast, magnetite nanoparticles prepared via the traditional chemical method exhibited an average size of 27.9 nm, as calculated employing the Debye–Scherrer formula.

### 3.3 Scanning electron microscope (SEM)

**Fig. 8:**
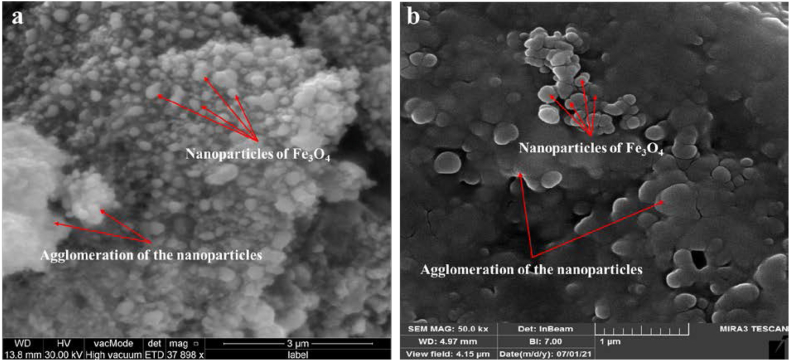
SEM image a) Myco-synthesized Fe_3_O_4_ NPs from growth medium b) the magnetite NPs prepared from conventional chemical method.

**Fig. 9:**
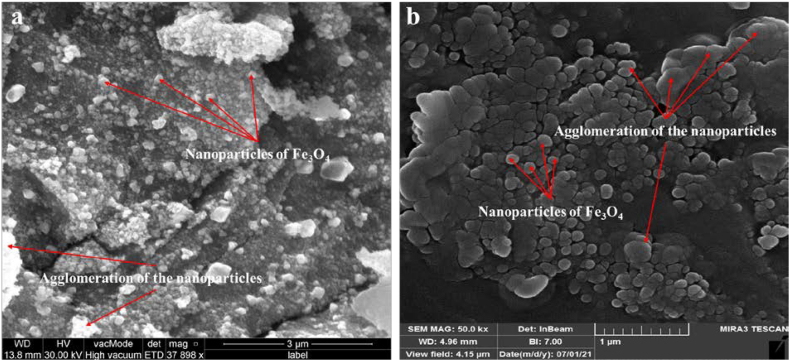
SEM image a) Myco-synthesized Fe_3_O_4_ NPs from aqueous extract b) the magnetite NPs prepared from conventional chemical method.

To describe the surface morphology of the biosynthesized Fe_3_O_4_ NPs, SEM examination was utilized [[63], [64], [65], [66], [67], [68]]. Fig. 8, Fig. 9 display the photos of the magnetite nanoparticles produced by myco-synthesized and conventional chemical method. As can be observed, most of the Fe_3_O_4_ NPs produced via myco-synthesized are on the nanoscale and have a uniformly hexagonal shape (Fig. 8, Fig. 9a). In addition, the Fe_3_O_4_ NPs have a somewhat agglomerated structure due to synthesized nanoparticles by biological methods. Agglomeration or aggregation occurs due to the high attraction that biosynthetic NPs have for one another, which contributes to the increased surface area that the myco-synthesized NPs possess. The magnetite nanoparticles produced by the conventional chemical process do not have a uniform distribution, and their diameters are enormous (Fig. 8, Fig. 9b). In addition, the magnetite nanoparticles produced using conventional chemical processes resulted in a highly agglomerated product [63,66,69].

The formation of Fe_3_O_4_ NPs nanoparticles relies on several growth parameters. These growth factors include the biomass of fungal species, concentration of fungal extract, type of growth media, concentration of salt, amount of time spent growing or reacting, temperature, and pH of the solution. Consequently, proper optimization of these development components is essential for NPs to attain the smaller size and uniform shapes needed for maximal manipulation and demand. In this study, the growth parameter used are the optimal growth parameters [[70], [71], [72]].

### 3.4 Fourier transform infrared (FTIR)

**Fig. 10:**
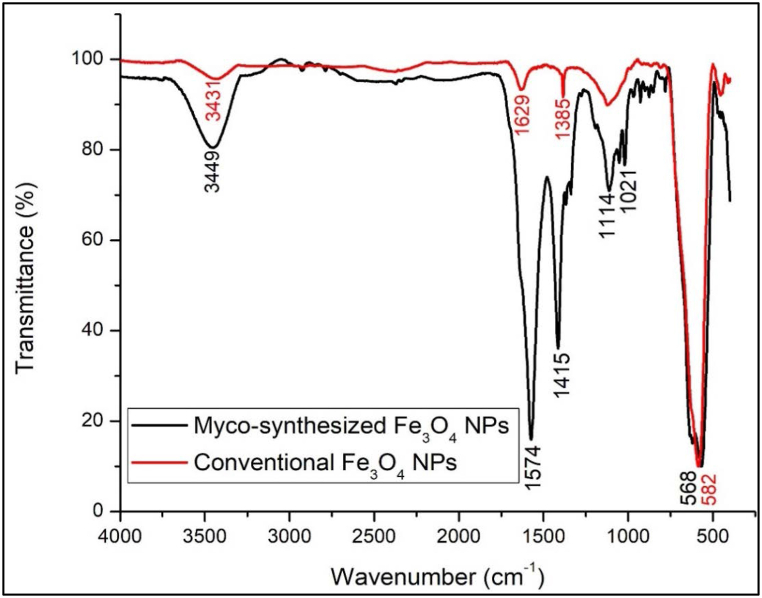
FTIR spectra of Myco-synthesized Fe_3_O_4_ NPs from the growth medium, and the magnetite NPs prepared from the conventional chemical method.

**Fig. 11:**
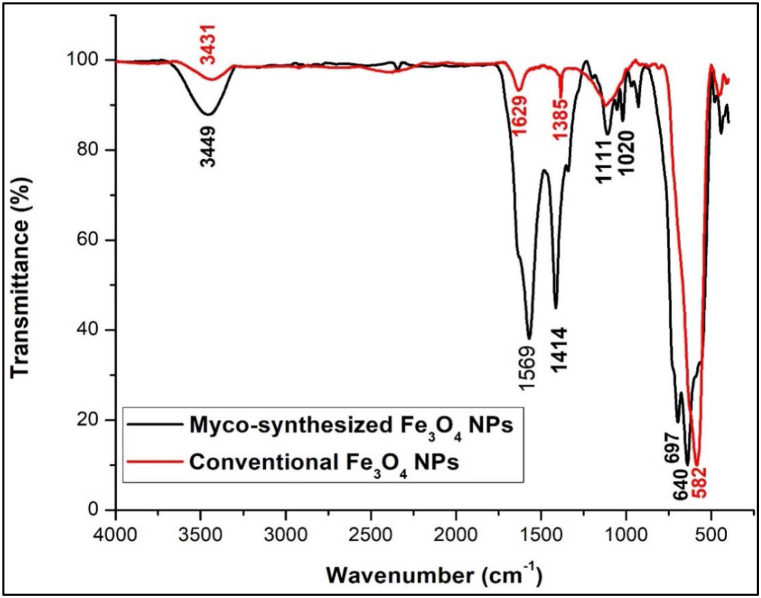
FTIR spectra of Myco-synthesized Fe_3_O_4_ NPs from aqueous extract and the magnetite NPs prepared from the conventional chemical method.

One of the accurate methods for investigation of the transmission bands of bimetallic magnetic systems is analyzed by FTIR spectroscopy, which is a method that can differentiate between the primary phases of iron oxide with a high degree of precision [[73], [74], [75]]. Fig. 10, Fig. 11 present the FT-IR spectra of Fe_3_O_4_ nanoparticles that were produced using the myco-synthesized and conventional chemical methods. An OH-stretching vibration can be seen at 3449 cm^−1^ from both myco-synthesized Fe_3_O_4_ NPs. This vibration receives contributions from symmetrical and asymmetrical modes of the O–H bonds related to the surface iron atoms. The identical peak was observed again within the magnetite NPs that were made using the conventional chemical process that was noticeably narrower. The large surface area of the myco-synthesized Fe_3_O_4_ NPs is most likely to blame for the broadness of the OH group found on the surface of the NPs. As a result, there are more sites on the surface of the NPs. The findings of this inquiry from those studies were not like others in other investigations, which may be because a different fungal species (*A.elegans*) was used in this investigation [[75], [76], [77]]. The second peak, which indicated the OH bending vibration, was 1574, 1569, and 1629 cm^−1^ from myco-synthesized Fe_3_O_4_ NPs from the growth medium, aqueous extract, and conventional chemical methods, respectively. These values were compatible with previously reported FTIR spectra for spinel Fe_3_O_4_ [78,79]. Also, the peak at 1629 cm^−1^ may indicate the presence of Fe–O. It may be deduced from the absorption bands at 1415 cm^−1^, 1414 cm^−1^, 1385 cm^−1^ from myco-synthesized Fe_3_O_4_ NPs from the growth medium solution, aqueous extract and conventional chemical methods that asymmetric stretching of CO originating from the acid group was present, respectively. For both myco-synthesized Fe_3_O_4_ NPs and conventional chemical methods, the stretching vibration mode associated with the metal-oxygen Fe–O bonds in Fe's crystalline lattice is thought to be responsible for the strong peak observed at 568 cm^−1^, 640 cm^−1^, and 582 cm^−1^, respectively. They are quite noticeable in the structures of all spinel minerals, especially ferrites [[80], [81], [82]].

### 3.5 Transmission electron microscope (TEM)

**Fig. 12:**
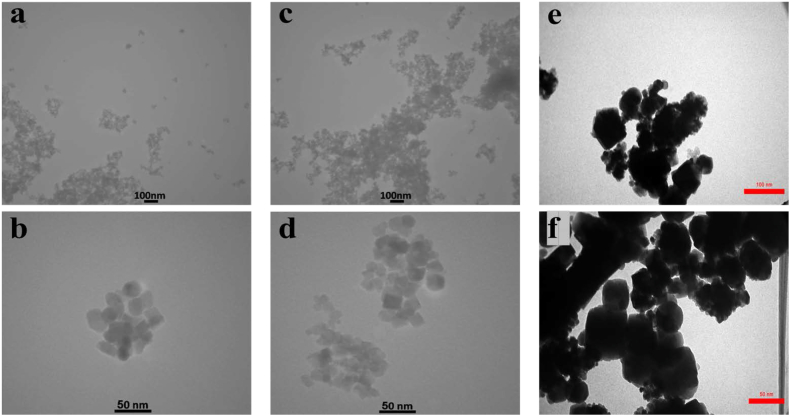
TEM image for (a and b) Myco-synthesized Fe_3_O_4_ NPs from the growth medium, (c and d) Myco-synthesized Fe_3_O_4_ NPs from aqueous extract, and (e and f) the magnetite NPs prepared from conventional chemical method.

**Fig. 13:**
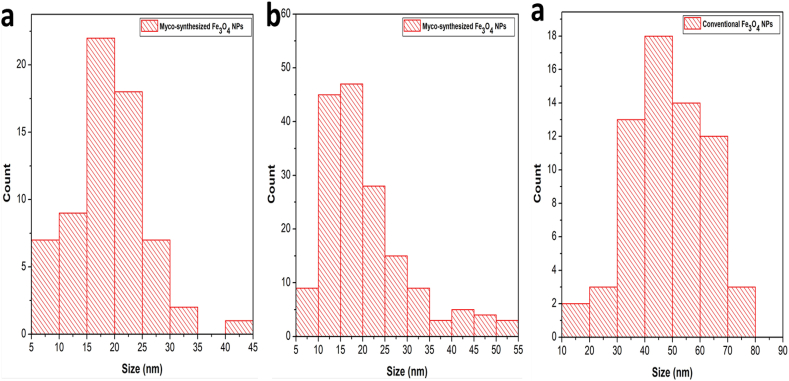
A particle size distribution histogram determined from the TEM images a) Particle size distribution of Myco-synthesized Fe_3_O_4_ NPs from the growth medium b) Particle size distribution of Myco-synthesized Fe_3_O_4_ NPs from aqueous extract c) Particle size distribution of the magnetite NPs prepared from conventional chemical method.

An investigation of the shape and distribution of myco-synthesized Fe_3_O_4_ NPs and conventional Fe_3_O_4_ NPs was carried out using transmission electron microscopy (TEM). Both myco-synthesized Fe_3_O_4_ NPs (Fig. 12a–d), and conventional Fe_3_O_4_ NPs (Fig. 12e and f) seemed to be composed of hexagonal particles when seen using typical transmission electron micrographs. The particle size histogram of myco-synthesized Fe_3_O_4_ NPs from the growth medium (Fig. 13a) demonstrated particle diameters ranging from 5 to 55 nm, with an average size of 19.2 nm. The particle size histogram of myco-synthesized Fe_3_O_4_ NPs from aqueous extract (Fig. 13b) demonstrated particle diameters ranging from 5 to 45 nm, with an average size of 22.2 nm. While the particle size histogram of conventional Fe_3_O_4_ NPs (Fig. 13c) demonstrated particle diameters ranging from 10 to 80 nm, mostly about 60 nm). The findings of this study agree with previous studies [77,83]. It has been shown that the Fe_3_O_4_-NPs produced by the myco-synthesized approach are considerably more stable and smaller than those produced by the conventional chemical process. When the size of the Fe_3_O_4_-NPs is reduced, the surface area increases, which results in an increased number of capping agents around the myco-synthesized Fe_3_O_4_-NPs. The presence of additional capping agents surrounding myco-synthesized Fe_3_O_4_ nanoparticles enhances both their stability and zeta potential value. Furthermore, the substantial surface area of Fe_3_O_4_ nanoparticles offers several advantages, including the ability to efficiently detect molecular interactions and facilitate targeted biomolecule interactions. In essence, myco-synthesized Fe_3_O_4_ nanoparticles exhibit excellent stability and hold significant promise for a wide range of applications, including but not limited to biosensors, drug delivery, magnetic resonance imaging (MRI), cancer diagnostics, electrical devices, physical instruments, and sensor systems for monitoring pollutant gases [84].

### 3.6 Vibrating-sample magnetometer (VSM)

**Fig. 14:**
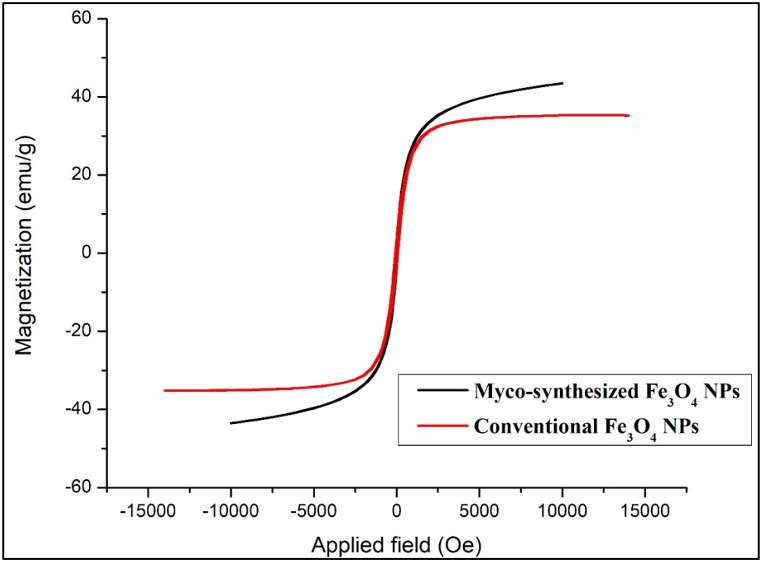
VSM analysis for Myco-synthesized Fe_3_O_4_ NPs from the growth medium and the magnetite NPs prepared from the conventional chemical method.

**Fig. 15:**
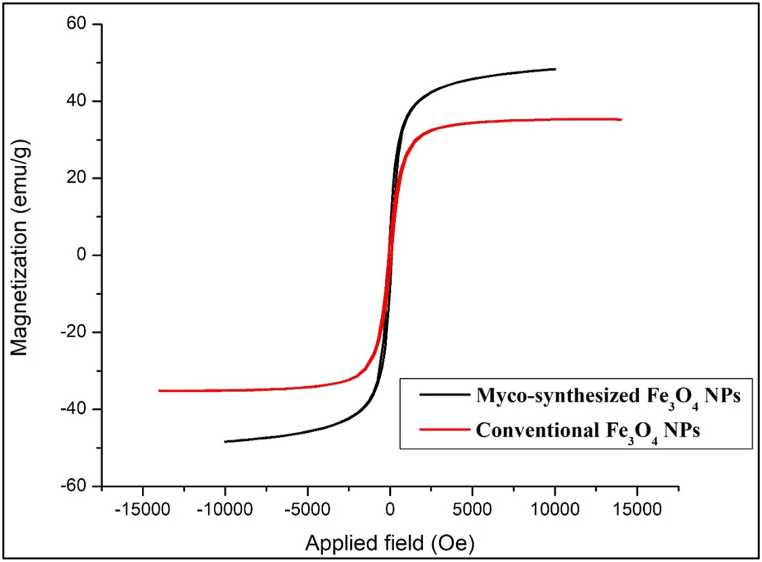
VSM analysis for Myco-synthesized Fe_3_O_4_ NPs from aqueous extract and the magnetite NPs prepared from the conventional chemical method.

To study synthesized Fe_3_O_4_ magnetic behavior, the magnetic properties of the NPs were measured at room temperature with a vibrating sample magnetometer (VSM) [85]. Fig. 14, Fig. 15 show the hysteresis loops of the samples. The saturation magnetization was 43.5 emu/g and 48.3 emu/g for myco-synthesized Fe_3_O_4_ NPs from the growth medium and aqueous extract, respectively. While, the saturation magnetization of the magnetite NPs prepared from the conventional chemical method was 35.4 emu/g as shown in Fig. 14, Fig. 15. From both myco-synthesized Fe_3_O_4_ NPs, no reduced remanence and coercivity were detected, indicating that myco-synthesized Fe_3_O_4_ NPs are superparamagnetic. The findings obtained from this study about saturation magnetization of the nanoparticle were more than green synthesized Fe_3_O_4_ NPs from *Rhus coriaria* extract that was confirmed in a previous study[86], which states that the magnetization value is 41 emu/g.

### 3.7 Thermogravimetric analysis

**Fig. 16:**
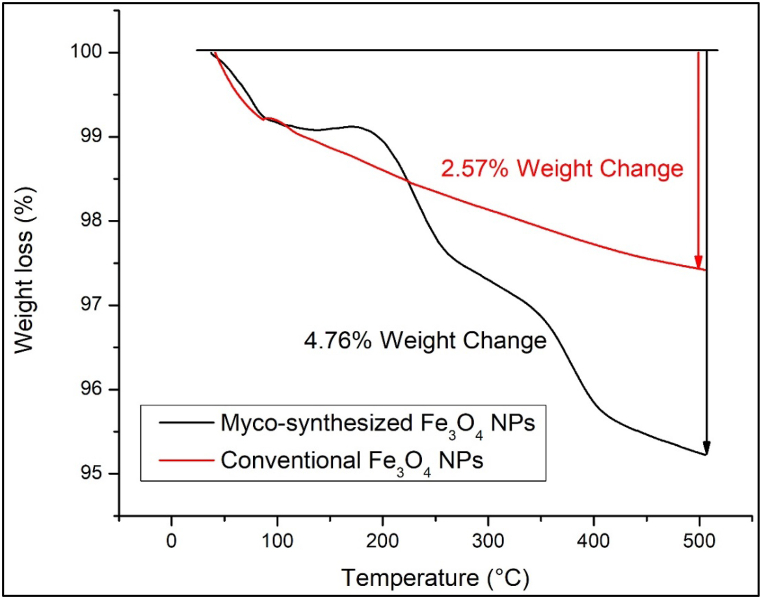
TGA analysis for Myco-synthesized Fe_3_O_4_ NPs from the growth medium and the magnetite NPs prepared from the conventional chemical method.

**Fig. 17:**
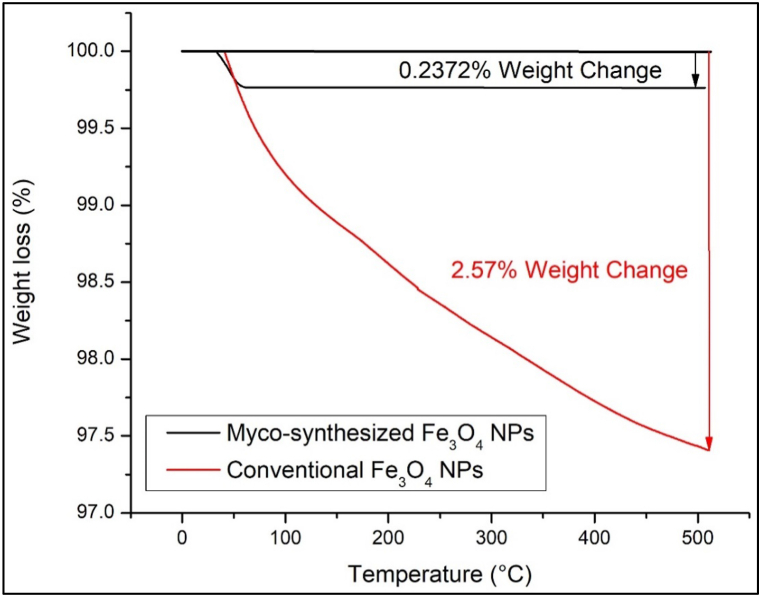
TGA analysis for Myco-synthesized Fe_3_O_4_ NPs from aqueous extract and the magnetite NPs prepared from the conventional chemical method.

Thermogravimetric analysis is a technique used to track alterations in the physical and chemical properties of materials as a function of temperature [[87], [88], [89], [90]]. In the case of myco-synthesized Fe_3_O_4_ nanoparticles and magnetite nanoparticles prepared through a conventional chemical approach, TGA was conducted over a temperature range of 0–500 °C, as depicted in Fig. 16, Fig. 17. The initial weight loss observed for the magnetite nanoparticles under 100 °C, is attributed to moisture content. Subsequently, decomposition occurs around 318 °C, attributed to the breakdown of protein chains, with capping agent decomposition occurring in the range of 150–400 °C, in accordance with previous findings [91]. In Fig. 16 shows myco-synthesized Fe_3_O_4_ nanoparticles experienced a weight loss of 4.76 % when subjected to a temperature of 500 °C, while magnetite nanoparticles prepared through the conventional chemical method exhibited a 2.57 % reduction in weight under the same temperature conditions. The large weight loss of myco-synthesized Fe_3_O_4_ NPs from the growth medium after being heated to 250 °C occurred due to capping agent decomposition [86,91]. However, the weight of myco-synthesized Fe_3_O_4_ NPs from aqueous extract was decreased by 0.24 % at 500 °C because of the evaporation of physical water inside the nanoparticles [92]. Based on the TGA analysis, myco-synthesized Fe_3_O_4_ NPs from the aqueous extract were stronger when exposed to high temperatures than the magnetite NPs prepared from the conventional chemical method at the same temperature.

### 3.8 Differential Scanning Calorimetry (DSC)

**Fig. 18:**
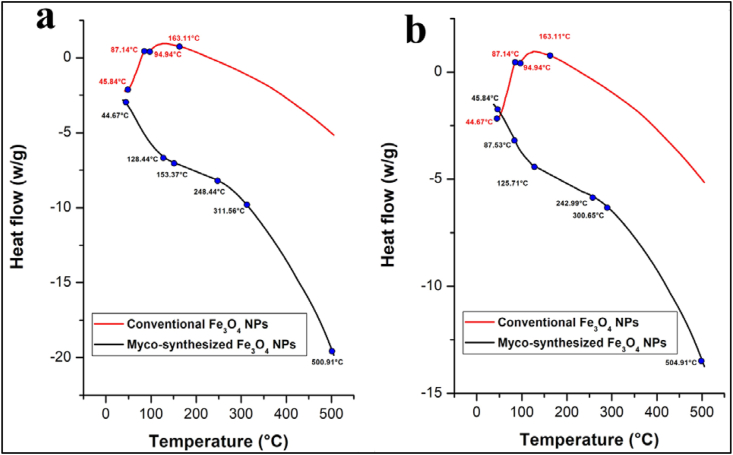
DCS curve for a) Myco-synthesized Fe_3_O_4_ NPs from the growth medium b) Myco-synthesized Fe_3_O_4_ NPs from aqueous extract and the magnetite NPs prepared from conventional chemical method.

Differential Scanning Calorimetry, abbreviated as DSC, is a thermal analysis method that quantifies the heat exchange within a sample while subjecting it to a precisely controlled temperature program. This technique serves as a robust tool for assessing various material characteristics, including critical factors such as the glass transition temperature (Tg), melting and crystallization phenomena, specific heat capacity, curing mechanisms, purity levels, susceptibility to oxidation, and thermal stability [66,86]. DSC analysis for both myco-synthesized Fe_3_O_4_ NPs shown that the glass Transition temperature of myco-synthesized Fe_3_O_4_ NPs was about 300 °C it was very high compared to the glass Transition temperature of the magnetite NPs prepared from the conventional chemical which is 163 °C (Fig. 18ab). Additionally, increasing (Tg) improves handling characters, solubility, and reproducibility in the dissolution of solids and also increases entropy that led myco-synthesized Fe_3_O_4_ NPs to be amorphous nanoparticle [93,94].

### 3.9 Dynamic light scattering (DLS)

**Fig. 19:**
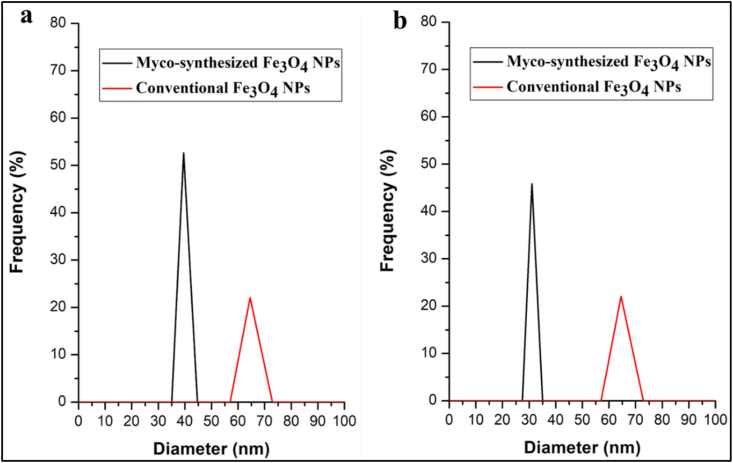
DLS analysis for Myco-synthesized Fe_3_O_4_ NPs a) Myco-synthesized Fe_3_O_4_ NPs from the growth medium b) Myco-synthesized Fe_3_O_4_ NPs from the aqueous extract and compared to the magnetite NPs prepared from conventional chemical method.

The DLS measurements reveal distinct nanoparticle sizes for different synthesis methods. Myco-synthesized Fe3O4 nanoparticles from the growth medium exhibit an average size of 39.5 nm (Fig. 19a), while those from the aqueous extract are smaller, measuring around 31 nm (Fig. 19b). In contrast, nanoparticles produced using the conventional chemical method are considerably larger, with an average size of 60 nm (Fig. 19ab). These variations in nanoparticle size can be attributed to the unique conditions and mechanisms involved in each synthesis approach. The growth medium may have facilitated the formation of smaller particles due to specific biochemical factors. In contrast, the chemical method likely led to larger nanoparticles through controlled nucleation and growth processes. The results agree with previous studies [77,83]. The nanoparticle sizes obtained from DLS measurements appear larger than those determined via TEM analysis. This dissimilarity arises from the influence of phytochemical molecules, which effectively augment the particle size. Additionally, the variation in conditions during the two analysis methods contributes to the contrast in size measurements. Specifically, the TEM analysis involves dried particles, whereas DLS analyzes particles suspended in aqueous solutions. Consequently, the hydrodynamic size of nanoparticles in colloidal suspension consistently exceeds the sizes determined by TEM, primarily due to the presence of adsorbed aqueous molecules [83,95].

### 3.10 Zeta potential

**Fig. 20:**
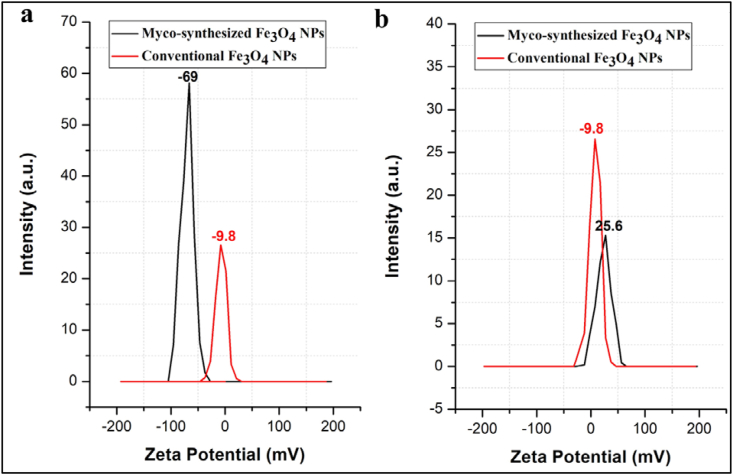
Zeta Potential analysis. a) Myco-synthesized Fe_3_O_4_ NPs from the growth medium b) Myco-synthesized Fe_3_O_4_ NPs from aqueous extract, both Myco-synthesized Fe_3_O_4_ NPs are compared to the magnetite NPs prepared from conventional chemical method.

Zeta potential analysis is a technique used to measure the surface charge or electrokinetic potential of particles suspended in a liquid medium, typically in colloidal systems. It provides insights into the stability and behavior of colloidal dispersions, such as nanoparticles, colloidal particles, and macromolecules. Nanoparticles possessing zeta potentials ranging from >^-^10 mV to <^+^10 mV have a neutral charge. Conversely, Nanoparticles possessing zeta potentials ranging from >^-^30 mV to <^+^30 mV are categorized as strongly cationic and strongly anionic, respectively [96]. The zeta potential measurement for Fe_3_O_4_ nanoparticles produced in this work from the growing media by myco-synthesis was found to be ^-^69 mV (Fig. 20a). The surface of the nanoparticles had a negative charge, which helped keep them stable and prevented the aggregation of iron particles. In comparison, the magnetite nanoparticles made using the traditional chemical approach had a zeta potential value of ^-^9.8 mV (Fig. 20ab). Therefore, it can be inferred from these findings that the myco-synthesized Fe3O4 nanoparticles are more stable than the magnetite nanoparticles made by a traditional chemical method.

## 4 Conclusions

In conclusion, this study employed a myco-synthesized approach to fabricate Fe_3_O_4_ nanoparticles and systematically compared them with Fe3O4 nanoparticles produced through a conventional chemical method. Generally, Myco-synthesized Fe_3_O_4_ NPs from the growth medium showed a quite better characterizations compared Myco-synthesized Fe_3_O_4_ NPs from the aqueous solution medium due to the reducing agents were screated into the medium to obtained the nutrients. Several key findings emerged from the microstructure tests, shedding light on the distinct characteristics and advantages of myco-synthesized Fe3O4 nanoparticles, which were smaller in size, hexagonal in shape, more stable, and also have higher magnetization values compared to Fe_3_O_4_ nanoparticles produced through a conventional chemical method.

## Data availability statement

Available in the manuscript.

## CRediT authorship contribution statement

**Renjbar Muksy Mhammedsharif:** Visualization, Validation, Supervision, Software. **Parwin Jalal Jalil:** Conceptualization, Validation, Visualization. **Nzar Piro:** Project administration, Resources. **Ahmed Mohammed:** Funding acquisition, Data curation, Conceptualization. **Peyman K. Aspoukeh:** Conceptualization, Investigation, Methodology.

## Declaration of competing interest

The authors declare that they have no known competing financial interests or personal relationships that could have appeared to influence the work reported in this paper.
